# Capturing 3D Chromatin Maps of Human Primary Monocytes: Insights From High-Resolution Hi-C

**DOI:** 10.3389/fimmu.2022.837336

**Published:** 2022-03-03

**Authors:** Yu Xia, Xiaowen Liu, Wenli Mu, Chunyan Ma, Laicheng Wang, Yulian Jiao, Bin Cui, Shengnan Hu, Ying Gao, Tao Liu, Huanxin Sun, Shuai Zong, Xin Liu, Yueran Zhao

**Affiliations:** ^1^ Department of Central Laboratory, Shandong Provincial Hospital, Cheeloo College of Medicine, Shandong University, Jinan, China; ^2^ Department of Central Laboratory, Shandong Provincial Hospital Affiliated to Shandong First Medical University, Jinan, China; ^3^ Department of Clinical Laboratory, The First Affiliated Hospital of Shandong First Medical University & Shandong Provincial Qianfoshan Hospital, Jinan, China; ^4^ Bioinformation Center, Annoroad Gene Technology (Beijing) Co., Ltd., Beijing, China

**Keywords:** 3D chromatin maps, primary monocytes, Hi-C, HLA, CD16

## Abstract

Although the variation in chromatin architecture during adaptive immune responses has been thoroughly investigated, the 3D landscape of innate immunity is still unknown. Herein, chromatin regulation and heterogeneity among human primary monocytes were investigated. Peripheral blood was collected from two healthy persons and two patients with systemic lupus erythematosus (SLE), and CD14^+^ monocytes were selected to perform Hi-C, RNA-seq, ATAC-seq and ChIP-seq analyses. Raw data from the THP1 cell line Hi-C library were used for comparison. For each sample, we constructed three Hi-C libraries and obtained approximately 3 billion paired-end reads in total. Resolution analysis showed that more than 80% of bins presented depths greater than 1000 at a 5 kb resolution. The constructed high-resolution chromatin interaction maps presented similar landscapes in the four individuals, which showed significant divergence from the THP1 cell line chromatin structure. The variability in chromatin interactions around HLA-D genes in the HLA complex region was notable within individuals. We further found that the CD16-encoding gene (*FCGR3A*) is located at a variable topologically associating domain (TAD) boundary and that chromatin loop dynamics might modulate CD16 expression. Our results indicate both the stability and variability of high-resolution chromatin interaction maps among human primary monocytes. This work sheds light on the potential mechanisms by which the complex interplay of epigenetics and spatial 3D architecture regulates chromatin in innate immunity.

## Introduction

Chromatin is hierarchically packaged into the nucleus of higher eukaryotes to organize the three-dimensional (3D) genome structure (1), which is responsible for precise transcriptional regulation by facilitating or restricting regulatory element interactions (2). Over the last decade, the 3D genome architecture associated with cell fate and function under both physiological and pathological conditions has garnered much attention and has been intensively investigated through advanced techniques (3, 4). Chromosome conformation capture (3C) technologies, from 3C, 4C, 5C to chromatin interaction analysis with paired-end tag sequencing (ChIA-PET) and Hi-C, have become increasingly appreciated for their ability to facilitate the comprehensive identification of genome-wide contact frequencies (5, 6), which can reveal chromatin organization features, including A/B compartments, TADs and loops (7, 8). Evidence demonstrating the role of the 3D genome organization in governing long-range regulatory interactions is now available (9, 10), and such work provides a new blueprint for investigating the influence of spatiotemporal changes in 3D architecture during normal evolution and disease occurrence (11).

Chromatin interactions play a fundamental role in establishing and maintaining the functions of immune cells during development, differentiation and activation in autoimmune diseases (12, 13). An analysis of genome organization performed in 17 human primary hematopoietic cell samples by promoter capture Hi-C (PCHi-C) indicated that the promoter interactomes are highly cell-type specific (14). Much evidence has validated the concept that transient changes in the genomic architecture in human B cells and CD4^+^/CD8^+^ T cells require the recruitment of specific lineage-defining transcription factors, chromatin remodelers, and histone modifiers to modulate gene transcription and mediate B and T cell lineage commitment (15–17). While a series of studies on chromatin 3D organization have focused on adaptive immune responses, little is known about its role in innate immunity. The issue associated with 3D structure in innate immune cells that has been investigated is long-range looping interactions during differentiation from monocyte precursors (THP1 cell line) to mature macrophages (18, 19), which suggested the possibility that the spatial 3D structure might regulate innate immunologic processes. In general, almost all of the available Hi-C maps have been displayed in cultured immune cells. In addition, recent research combining associated genetic variants identified from Genome-wide Association Studies (GWASs) with 3D structures observed in different immune cell physiological states has revealed potential regulatory connections of these noncoding region variants related to the pathogenesis of autoimmune diseases (20, 21); this work has yielded pivotal insights into how autoimmunity is triggered by susceptible polymorphisms (22).

To date, chromatin architecture variation during adaptive immune response has been thoroughly investigated, although few previous research on Hi-C had been performed in primary monocytes, there still lacks a high-resolution 3D landscape in innate immunity and analysis of individual difference. SLE, as the examined autoimmune condition, is a highly heterogeneous autoimmune disease characterized by the production of numerous autoantibodies and chronic inflammation (23). SLE can systematically and severely affect multiple organs, including central nervous system and peripheral nervous system. Therefore, the present study was designed to first present high-resolution chromatin interaction maps of human primary monocytes and then to make further efforts to elucidate the immunological heterogeneity of the 3D genome structure. We hope to provide insight into the functional regulation of monocytes in innate immunity by comparing healthy controls and SLE patients to identify significant epigenetic profiles associated with chromatin accessibility as well as histone modification patterns correlated with the transcriptional profiles of human primary monocytes, as evidenced by integrated datasets from assay for transposase-accessible chromatin sequencing (ATAC-seq), chromatin immunoprecipitation with sequencing (ChIP-seq), and RNA sequencing (RNA-seq) analyses.

## Materials and Methods

### Antibodies and Reagents

The antibodies and reagents used in this study were as follows: Alexa Fluor^®^ 488 mouse anti-human CD14 was purchased from BD Pharmingen™ (561706). 7-Amino-actinomycin D (7-AAD) was purchased from BD Pharmingen™ (559925). Protease inhibitors were purchased from Sigma (P8340-5 ml). Biotin-14-dCTP and Proteinase K (Fungal) were purchased from Invitrogen (19518-018; 25530-031). DNA polymerase I, large (Klenow) fragment, was obtained from NEB (M0210S). T4 DNA ligase, T4 DNA polymerase and T4 DNA ligation buffer were all purchased from NEB (M0202L; M0203L; B0202).

### Study Subjects

SLE patients (n=2, SLE-1; SLE-2) were recruited from Shandong provincial hospital affiliated to Shandong university. All patients with SLE met the revised diagnostic criteria of the American College of Rheumatology (1997), and other systematic or autoimmune diseases were excluded (24). None of the patients had been using systemic or topical medication before, and all were characterized by high ANA and dsDNA antibody levels and low-level clinical symptoms during sample collection. Disease activity was measured using the SLE Disease Activity Index scoring system. The control population (n=2, CTR-1; CTR-2) consisted of unrelated individuals matched for ethnicity, age, and sex. The four donors were all 25 years old. Valid informed consent was obtained from each participant. The study design conformed to the ethical guidelines and was approved by the ethics committee of Shandong Provincial Hospital affiliated to Shandong first medical university.

### Preparation of Monocytes

From each donor, a 150 ml blood sample was collected, and 30 ml of fresh peripheral blood was collected at each sampling time to keep the cells as active as possible. Ficoll-Paque (GE 17-1440-02) density gradient centrifugation was used to separate peripheral blood mononuclear cells (PBMCs) within 2 h. The PBMCs were stained and incubated with an antibody cocktail (7-AAD and Alexa Fluor^®^ 488 mouse anti-human CD14) in darkness at room temperature for 20 min. After incubation, the cells were washed twice with 1% heat-inactivated fetal bovine serum diluted in PBS for fluorescence-activated cell sorting (FACS). The cells were sorted by flow cytometry using a FACS Aria III system (Becton Dickinson), and approximately 5 million cells per sample were obtained and defined as CD14^+^ monocytes. The activity of the selected CD14^+^ primary monocytes reached greater than 95%.

### RNA-Seq and Data Analysis

The RNA-seq of monocytes was performed *via* the Smart-Seq2 method. Briefly, samples were collected in tubes with lysis components and ribonuclease inhibitors. An Oligo-dT primer was introduced to the reverse transcription reaction for first-strand cDNA synthesis, followed by PCR amplification to enrich cDNA and a MagBeads purification step to clean up the product. Then, the cDNA product was checked with a Qubit^®^ 3.0 Fluorometer and Agilent 2100 Bioanalyzer to verify the expected product with a length of approximately 1~2 kb. Next, the cDNA was randomly sheared by ultrasonication according to the Illumina library preparation protocol, which included DNA fragmentation, end repair, 3’ end A-tailing, adapter ligation, PCR amplification and library validation. After library preparation, the PerkinElmer Lab ChIP^®^ GX Touch system and the Step OnePlus™ Real-Time PCR system were used for library quality inspection. Qualified libraries were then loaded on the Illumina HiSeq platform for PE150 sequencing. Sequencing data were mapped to the human reference genome (ucsc.hg19) using HISAT2 v2.1.0. fragments per kilobase of transcript per million mapped reads (FPKM) values for each gene were calculated. RNA-seq data were shown in **Supplementary Data Sheet 1** and the quality control of RNA-seq was in **Figure S1**.

### ATAC-Seq and Data Analysis

Approximately 50,000 living cells were used for each library preparation. The cells were lysed in lysis buffer to obtain nuclei, and the TruePrep™DNA Library Prep Kit V2 for Illumina (Vazyme Biotech) was used to construct transposase-treated libraries. The mass concentration and molar concentration of the libraries were determined with a Qubit 3.0 Fluorometer and the StepOnePlus™ Real-Time PCR system, respectively, and the lengths of the inserted fragments were determined with an Agilent HS 2100 Bioanalyzer. Qualified libraries were sequenced on the Illumina HiSeq platform in paired-end 150 bp mode. The data were mapped to the human reference genome (ucsc.hg19) with Bowtie2 (version 2.2.3), and binding sites were identified by using MACS2 (version 2.1.1) with the following parameters ‘-q 0.05 –nomodel –extsize 150 –keep-dup all –call-summits’. ATAC-seq data were shown in **Supplementary Data Sheet 2** and the quality control of ATAC-seq was in **Figure S2**.

### ChIP-Seq and Data Analysis

Nuclear extracts from approximately one million cells and chromatin were sheared to an average size of 200 bp with a sonicator (Bioruptor Pico, Diagenode). Then, the samples were immunoprecipitated with 2.5 μg of H3K27me3 (ab6002), anti-H3K4me3 (ab39915) and anti-H3K27Ac (ab39133) pAbs antibodies. After incubation at 4°C overnight, the antibodies were recovered with 25 μl of Protein A/G magnetic beads (Millipore 16-663). After reverse crosslinking, ChIP-ed DNA was extracted with a MinElute Reaction Cleanup Kit (Qiagen 28206). Purified DNA from H3K27me3, H3K27Ac and H3K4me3 ChIP assays was adapter ligated and PCR amplified for sequencing on the HiSeq2000 platform using a TruSeq DNA Library Prep Kit (Illumina). After sequencing, reads were quality-filtered according to the Illumina pipeline. The data were aligned to the human reference genome (ucsc.hg19) with Bowtie2 (version 2.2.3), and binding sites were identified with MACS2 (version 2.1.1). ChIP-seq data were shown in **Supplementary Data Sheet 3** and the quality control of ChIP-seq was in **Figure S3**.

### Hi-C and Data Analyses

#### Hi-C Library

Approximately one million monocytes were crosslinked with 40 ml of a 1% formaldehyde solution at room temperature for 10 min, and 2.5 M glycine was added to quench the crosslinking reaction. The crosslinked cells were resuspended in 500 µl of ice-cold Hi-C lysis buffer and rotated at 4°C for 30 min. The nuclei were washed with 0.5 ml of restriction enzyme buffer, and the chromatin was solubilized with diluted SDS. After quenching the SDS with Triton X-100, overnight digestion with cutter restriction enzymes (400 units MboI) was performed at 37°C. The DNA ends were marked with biotin-14-dCTP, and blunt-end ligation of crosslinked fragments was performed. The proximal chromatin DNA was religated with a ligation enzyme, and the nuclear complexes were reverse crosslinked at 65°C. The DNA was purified, and biotin-C was removed from the nonligated fragment ends using T4 DNA polymerase. The fragments were sheared to sizes of 100-500 base pairs by sonication. The fragment ends were then repaired with a mixture of T4 DNA polymerase, T4 polynucleotide kinase and Klenow DNA polymerase. Biotin-labeled Hi-C samples were specifically enriched using streptavidin magnetic beads. The fragment ends were subjected to A-tailing with Klenow (exo-), and an Illumina paired-end sequencing adapter was then added with a ligation mixture. Finally, the Hi-C libraries were amplified *via* 8-10 cycles of PCR and sequenced on an Illumina HiSeq instrument in PE150 mode.

The raw sequence data of the THP1 cell line Hi-C library were downloaded from ENCODE (Encyclopedia of DNA Elements Project Consortium) from experiment ENCSR748LQF (https://www.encodeproject.org/experiments/ENCSR748LQF/), which included two replicates (ENCBS615XLU, assigned as THP1-1; ENCBS913QYS, assigned as THP1-2) and analyzed *via* the same pipelines.

#### Hi-C Data Mapping, Filtering and Generation of Contact Matrices

FASTQ files were firstly subjected to quality control with fastp (version 0.14) software before mapping stages. We used the integrated pipeline of HiC-Pro (V2.7.8) to process the data from the clean FASTQ files to obtain contact maps with default parameters (MIN_MAPQ=10; BOWTIE2_GLOBAL_OPTIONS = very-sensitive; BOWTIE2_LOCAL_OPTIONS = very-sensitive; BIN_SIZE = 20000, 40000, 150000, 500000, 1000000), which mainly included two-step mapping using Bowtie2 and binning to generate a genome-wide interaction map. The resulting contact matrices were normalized using iterative correction and eigenvector decomposition (ICED, MAX_ITER = 100, FILTER_LOW_COUNT_PERC = 0.02; FILTER_HIGH_COUNT_PERC = 0; EPS = 0.1) (25).

The loops were called by using HiCCUPS (8) with the default parameters, which examines each pixel in a Hi-C contact matrix and identifies those with enriched contact frequencies relative to local neighborhoods.

To calculate the average contact probability (Ps), we divided the genome into 1 M bins. For each distance (1 M, 2 M, 3 M, etc.), we used the observed interaction frequency to calculate the expected value *via* LOWESS fitting at the corresponding distance (26). The relative contact probability (RCP) was computed for each chromosome, and the insulation score was used to call TADs. The above analysis was performed with GENOVA package by functions of insulation_score and callTAD.

The multiHiCcompare packages was used for comparative analysis. Briefly, the interaction matrix was firstly performed fast loess normalization (fastlo function) and performed exact test-based difference detection among groups (hic exactTest function). The composite MD plot were plotted by MD_composite function.

The visualization of chromatin interactions were preformed with the help of washU Epigenome Browser (27) and hicrep package.

#### Resolution Analysis

For Hi-C resolution calculation, the whole genome was divided into bins of the same size (1 M, 500 k, 200 k, 100 k, 40 k, 20 k, 10 k, 5 k, or 1 k), and valid pairs were then used to determine the coverage of every bin. We sorted the bins in descending order according to the coverage depth of the 75^th^, 80^th^, and 90^th^ bins. At a specific resolution, when the minimum depth of the 80^th^ bin reached 1000, we considered the sequencing depth to have reached the same resolution, which was then used as the highest resolution. The results showed that the resolution reached 5 kb and could be used for loop analysis.

### Genotyping of the Human Leukocyte Antigen (HLA) Region

About 200 ng sample of genomic DNA from each individual was sheared with a Biorupter (Diagenode, Belgium) to acquire 150~200 bp fragments. The ends of the DNA fragments were repaired, and an Illumina adapter was added (Fast Library Prep Kit, iGeneTech, Beijing, China). After constructing the sequencing library, the target regions were captured with an AI-HLA-Cap Enrichment Kit (iGeneTech, Beijing, China) and sequenced on an Illumina platform (Illumina, San Diego, CA) with 150 base pair paired‐end reads. Raw reads were filtered to remove low-quality reads by using FastQC, and the clean reads were then mapped to the reference sequences in the HLA dictionary and typed to generate HLA types for HLA-A, HLA-B, HLA-C, HLA-DPB1, HLA-DQB1, and HLA-DRB1 by using HLA-HD Software.

### Statistics

The Mann-Whitney test (unpaired) or the Wilcoxon matched-pair signed-rank test (paired) was performed to analyze the data. For multiple comparisons, analyses were performed using the Kruskal-Wallis test followed by Tukey’s test (unpaired) or Friedman’s test followed by Dunn’s test (paired). All analyses were performed with R 4.0. Statistical significance was reported as follows: *P < 0.05, **P < 0.01, ***P < 0.001.

## Results

### High-resolution Chromatin Interaction Maps of Monocyte Samples

The CD14^+^ primary monocytes from the peripheral blood of two SLE patients and two healthy donors were isolated and crosslinked to obtain high-resolution chromatin interaction maps. To determine whether the whole-genome chromatin conformation differed between the two groups, we constructed three MboI-digested Hi-C libraries for each sample. We combined the data from the replicates to obtain more than 1.2 billion valid reads in approximately 3 billion paired-end reads for the subsequent analysis. The valid rates for CTR-1, CTR-2, SLE-1 and SLE-2 were 67.02%, 63.33%, 70.39% and 68.35%, respectively (valid rate above 60%, **Table S1**).

Given the absence of high-resolution chromatin interaction maps of primary monocytes before, we used the Hi-C map of the human monocyte cell line THP1 as the reference for comparison. The data of THP1 were downloaded from the ENCODE database and processed consistently under the same pipeline as the four primary samples for the uniform standards for comparison. Due to the THP1 cell line was established from leukaemia patient, we especially checked the status of chromosomal rearrangement. In the genome wide cis and trans interactions (**Figure S4**), we could see that the normalized mean interactions within chromatin (cis) were always stronger than the interactions between chromatins, thus there was no large chromosomal rearrangement. However, the strengthened trans-interaction between the left end of 9p and right end of 11q (**Figures S4C, 4D**) suggested a proportion of chromosome rearrangement, and it might influence corresponding local chromatin structure.

**Figure 1:**
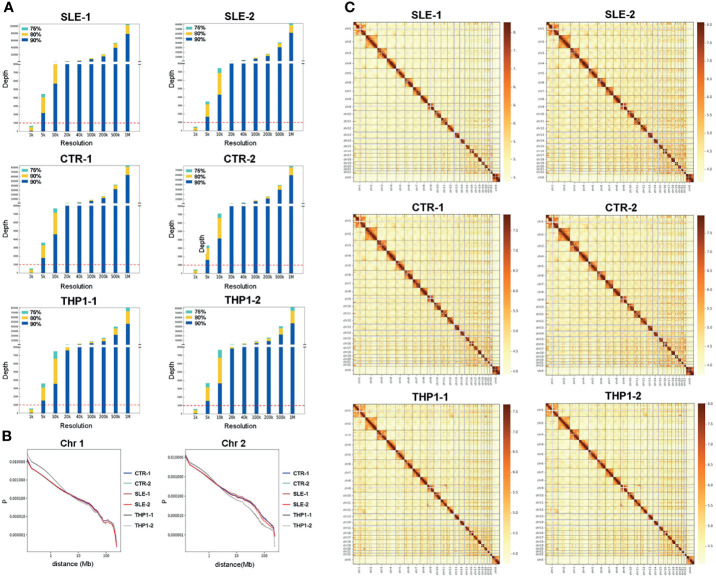
Chromatin interaction map of monocyte. The summary of high-resolution chromatin interaction maps of monocyte, including four primary monocyte samples of SLE-1, SLE-2, CTR-1, CTR-2 and two repeats of THP1. **(A)** Resolution analysis of the Hi-C data from six samples, the horizontal axis indicates the bin size of resolution, the vertical axis indicates the depth of reads. **(B)** The relative contact probability (RCP) in chromosome 1 and chromosome 2. **(C)** The heatmap of genome-wide chromatin interaction in 1Mb bin, the colors present logarithmic transformed normalized interaction value, Both the cis- (within chromosome) and trans- (inter chromosome) interactions are presented.

The general characteristics of the high-resolution chromatin interaction maps from the four primary monocyte samples and two repeats of the THP1 cell line are summarized in **Figure 1**. The resolution analysis showed that the results for the four primary individual samples reached more than 80% of bins at a depth greater than 1000 at a 5 kb resolution, which were similar with THP1 data (**Figure 1A**). These results indicated that our data were of high quality in both breadth and depth.

### High-Quality Chromatin Interaction Patterns of Monocyte Samples

We thoroughly explored the chromatin interaction patterns of the six monocyte samples at different levels. The relative contact probability (RCP) was computed to estimate the distance-dependent contact frequencies in chromosome level. The RCPs maps indicated the interaction frequency decayed with increasing distance in the six monocyte samples, while the curves for the four primary monocyte samples were largely in accord and differed slightly from those for the two THP1 repeats. Chromosome 1 and chromosome 2 are shown in **Figure 1B**, maps for other chromosomes are shown in **Figure S5**.

**Figure 2:**
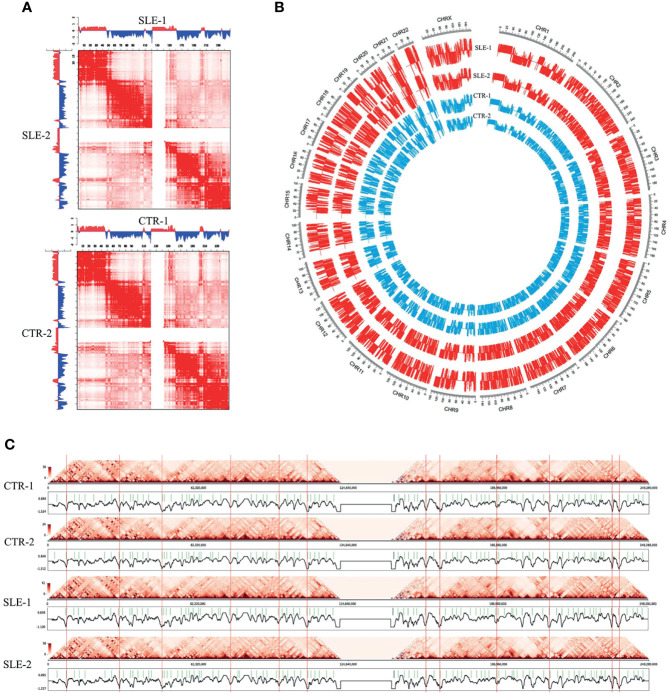
Landscapes of primary monocytes. The different levels of Hi-C maps of four primary monocyte samples (SLE-1, SLE-2, CTR-1 and CTR-2). **(A)** Heatmaps of normalized chromatin interactions (100 kb bin) in chromosome 1. The upper and lower triangles represented different samples, and to be specific, the first heatmap represents SLE-1 (upper) and SLE-2 (lower), and the second heatmap represents CTR-1 (upper) and CTR-2 (lower). **(B)** The genome-wide A/B compartment distribution of the four samples. **(C)** The insulation scores and TAD boundaries in chromosome 1 among the four samples.

Subsequently, we extracted the contact matrix of the six samples for further analysis and the genome compartment classification analysis was performed to category the A/B compartments (**Figure 2**). The normalized chromatin interactions of 100 kb resolution on chromosome 1 are shown in **Figure 2A**, whose upper and lower triangles represent different samples. The delta Hi-C matrix calculated by subtracting the genome-wide Hi-C interaction were shown in **Figure S6**. The circle diagram shown in **Figure 2B** displays the genome-wide A/B compartment distribution observed in the four samples. The TADs are defined as genomic clusters of chromatin interaction that act as both structural and functional units (28). We identified TADs using the clustering-based Hi-C domain finder (CHDF) method (29). **Figure 2C** describes the TADs on chromosome 1 in the four primary monocyte samples with the Hi-C interactive heat map.

The above results roughly shown hierarchical chromatin conformations and interaction patterns revealed by Hi-C maps in the four primary monocyte samples.

### Compartments and TADs Show No Significant Difference Among the Four Primary Monocyte Samples

To determine whether the chromatin conformations differed between primary cells and THP1 cell line or between the healthy and patient groups, we performed comparative analysis in different levels.

The correlation analysis of genome-wide A/B compartment exhibited high similarity among the four primary monocyte samples (**Figure S7**) with only a few regions presenting compartment switching between SLE patients and health individuals. The involved genes (353 genes) around switching regions and the KEGG enrichment analysis were presented in **Table S2**. In consist with the conservatism in genome compartmentalization, the interaction pattern shown in the heatmap of matrix also present high similarity among the four primary monocyte samples (**Figure 1C**). Subsequently, we extracted the contact matrix of the six samples for further analysis. **Figure 1C** shows the heat map of the genome-wide chromatin interactions in the 1 Mb bin, and both the cis (within-chromosome) and trans (between-chromosome) interactions are represented with logarithmic transformed normalized interaction values. The interaction pattern was distributed along the diagonal in the matrix maps, and the cis/trans ratio of our data was normal. In addition, we observed a similar organization pattern along the heatmap diagonal for the healthy and patient samples, and the contact frequency between each chromosome appeared unchanged according to the Hi-C data for the four primary monocyte samples.

Overall, the four peripheral blood monocyte samples from different individuals showed roughly similar landscapes of chromatin interaction frequencies (**Figures 1B, C**), interaction matrices (**Figure 2A**), A/B compartments (**Figure 2B**) and TAD boundaries (**Figure 2C**). The differences between two of the individuals in some genome regions were found to be relatively significant when the SLE-1 sample was compared to the CTR-2 sample (discussed later), but few consistent changes could be found between the SLE patients and healthy people. Our results indicated that the primary monocyte samples displayed a highly conserved structural pattern in terms of genome organization.

**Figure 3:**
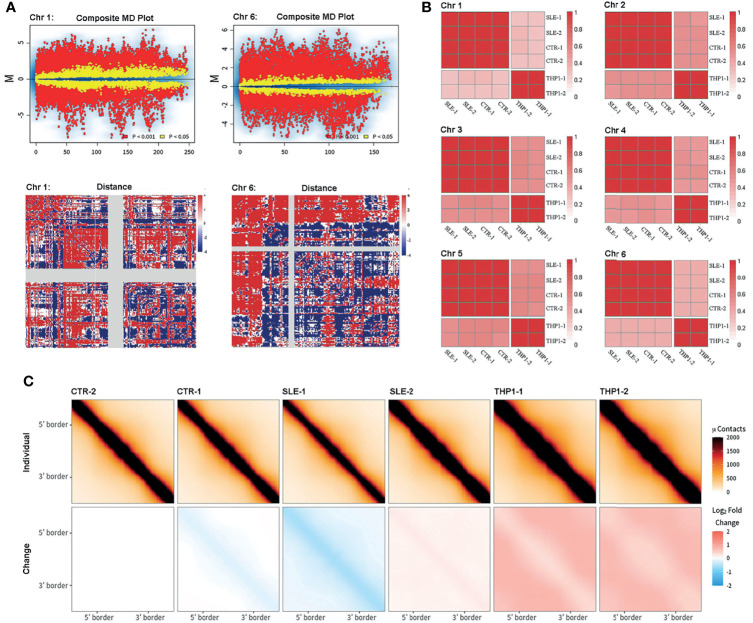
Comparison of Hi-C. Comparison of Hi-C among primary monocytes and THP1 by different methods. **(A)** The results from multiHiCcompare. The upper part displays the MD plot of comparison between four primary samples and two repeats of THP1, the lower part displays the difference matrix between primary monocytes and THP1. Here the results of chromosome 1 and chromosome 6 are presented. **(B)** The correlation matrix between the six samples. The pair-wised stratum-adjusted correlation coefficients (SCC) are calculated using hicrep package. The results of chromosome 1-6 are presented. **(C)** The aggregate peak analysis (APA) among the six samples. Here the we set CTR-2 as reference.

To more rigorously assess the homogeneity and divergence among individuals, we employed several comparative methods for Hi-C maps. The package of multiHiCcompare provided integrated algorithms to normalize and assess differences between Hi-C datasets, which could well remove biases across multiple datasets and detect decay of chromatin interaction frequencies. In the multiple-dataset comparative analysis, the primary samples differed significantly from the THP1 cells in the interaction matrix at a 100-kb resolution (chromosome 1 and chromosome 6 in **Figure 3A**, other chromosomes in **Figures S8** and**S9**, while the differences between SLE and control groups were mostly not significant.

The HiCRep package provided a stratum-adjusted correlation coefficient to assess the reproducibility of Hi-C data, and we used this method to do correlation analysis among the six samples (**Figure 3B** shows the correlation matrix among the six samples, and the results for chromosomes 1 to 6 are presented, other chromosome data are shown in **Figure S10**). According to the pairwise correlation coefficients, the similarly was greater than 0.97 within the THP1 repeats or within primary sample groups, but was greatly dropped between primary cells and THP1 repeats.

The aggregate peak analysis (APA) was designed to collect loop calls from Hi-C data and detect enriched pattern of loops. To access the difference of loops in genome wide, we chose the TAD boundary of the CTR-2 (the TAD boundary of CTR-2 exhibited the strongest effect among the four primary cells) sample as a reference to calculate the interaction enrichment of each sample. The results (**Figure 3C**) were represented as the difference compared to CTR-2 (minus the value of CTR-2). We found that the loop interaction was slightly decreased in the SLE-1 sample (blue) but significantly increased in the THP1 repeats (red), while the other samples showed little difference.

Collectively, all of these analyses indicated stability of chromatin interactions within individual samples and significant divergence between the THP1 cell line and primary monocytes at chromatin higher structure.

### Notable Variability of Chromatin Interactions Around the HLA Complex Region Within Individuals

**Figure 4:**
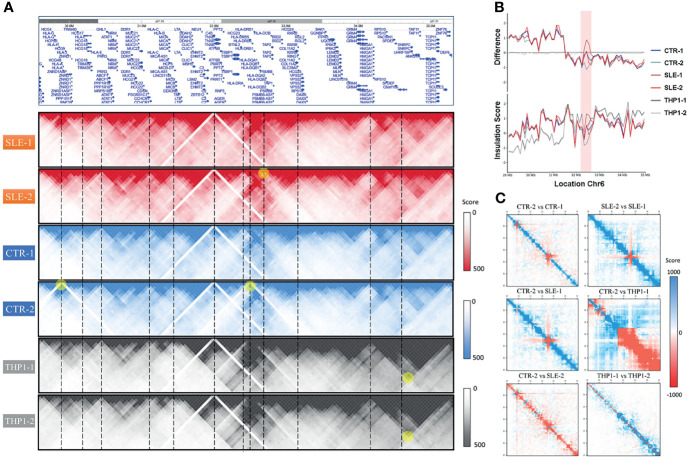
The polymorphism around HLA complex region. **(A)** The HLA complex region in chromosome 6 (upper) and the chromatin interactions of six samples around this region. **(B)** The value of insulation score of six samples (lower) and their difference to THP1-1 around HLA complex region (chr6: 29Mb-35Mb, hg19). **(C)** The heatmaps of pair-wised difference matrix.

Although we observed high conservation of the main structural features among the primary monocytes, the HLA complex region on chromosome 6 (6p21.3) presented notable variability in genome-wide cis-chromatin interactions (**Figure 4A**), which consists of genes belonging to MHC class I (classical genes: HLA-A, HLA-B and HLA-C; nonclassical genes: HLA-E, HLA-F and HLA-G), MHC class II (classical genes: HLA-DP, HLA-DQ and HLA-DR; nonclassical HLA genes: HLA-DM and HLA-DO) and MHC class III (complement and cytokine genes) (30). **Figure 4B** shows the insulation scores (lower) of the six samples around the HLA complex region (chr6: 29 Mb-35 Mb, hg19) and their differences between primary cells and the THP1 cell line (upper).

From the interactive matrix heat map, we deduced that in the p21.33 region, where HLA-C is located, THP1 cells presented fewer interactions than the primary samples, while THP1 presented more interactions at p21.32. Among primary monocytes, the variability was most significant around the HLA-D gene region of p21.32. Consistent with **Figure 4A**, in the p21.32 region, the SLE-1 sample showed the strongest chromatin interaction, and sample CTR-2 showed a relatively weak interaction. To show the differences between the samples, **Figure 4C** provides the heat maps of the pairwise difference matrix, where red represents enhancement, blue represents weakening, and the intensity of the color indicates the degree of the difference between samples. As shown by these comparison results, the four primary monocyte samples and the two THP1 cell line repeats showed large differences in certain regions of the polypeptides encoded by the HLA complex genes. Among the four primary cell lines, the difference between SLE-1 and CTR-2 was largest in this region, which was consistent with the results presented in **Figures 4A, B**.

### Potential Effects of Chromatin Interactions in the HLA Region

To investigate how chromatin organization influences the transcription and epigenetics of primary monocytes, in addition to Hi-C library preparation, the remaining collected monocytes of the four individuals were subjected to RNA-seq and ATAC-seq. Furthermore, three of the samples (SLE-1, SLE-2, CTR-2) underwent ChIP-seq analyses of H3K4me3, H3K27ac and H3K27me3.

**Figure 5:**
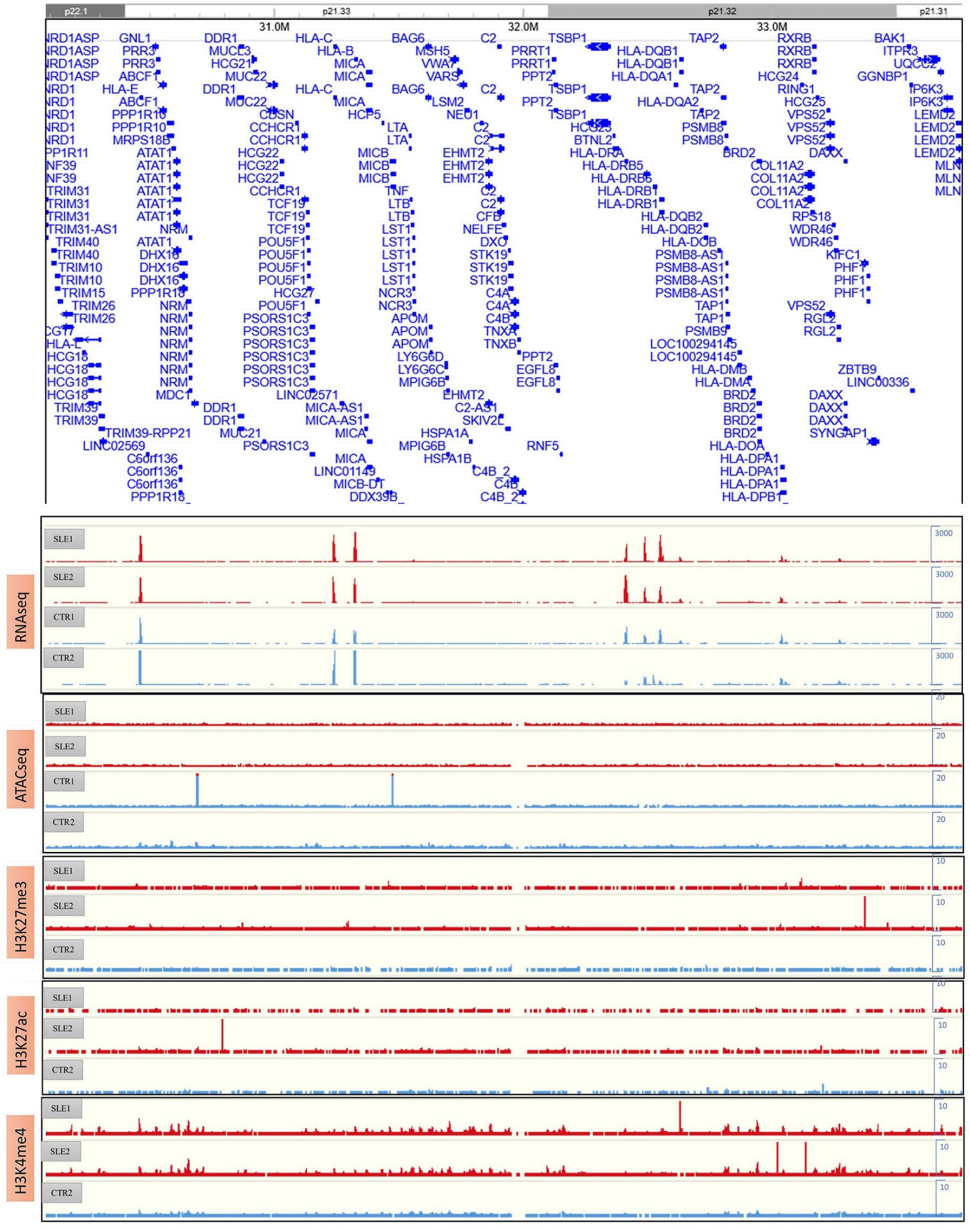
The results of RNA-seq, ATAC-seq and ChIP-seq of H3K27me3, H3K27ac and H3K4me4 around HLA complex region.

The RNA-seq, ATAC-seq and ChIP-seq results for HLA complex genes were presented in **Figure 5**. We found that the expression of HLA-DRB was higher in the SLE samples than in the CTR samples through RNA-seq; however, ATAC-seq and ChIP-seq did not provide consistent results, possibly because of the limited sample size and the high clinical heterogeneity and low quality of the samples. According to the RNA-seq results, HLA-DRB was strongly expressed in SLE-1 and weakly expressed in CTR-2, which was consistent with the chromatin interactions around this region, as shown in **Figure 4B**. The genotyping of the HLA regions (HLA-A, HLA-B, HLA-C, HLA-DPB1, HLA-DRB1 and HLA-DQB1) in the four primary samples was also performed (**Figure S11**), and all of these loci were heterozygous except for HLA-DRB1*15:01:01 and HLA-DQB1*06:02:01 in SLE-1. This result indicated that the high polymorphism of the DNA sequence might influence the 3D genome structure and then affect the regulation of associated gene expression patterns.

### Dynamic Chromatin Loops Might Regulate CD16 Expression

**Figure 6:**
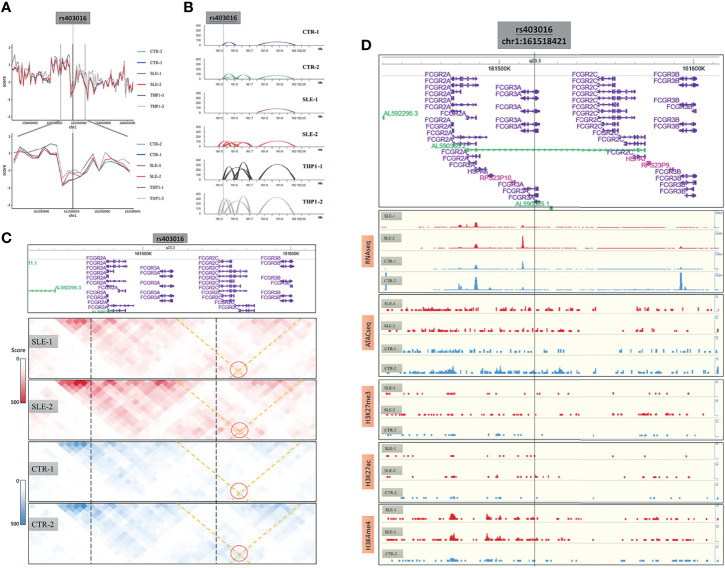
The 3D structure around CD16 coding gene. **(A)** The insulation score value of six samples around CD16 coding genes. **(B)** The loops of six samples around this region. **(C)** The chromatin interactions around this region. **(D)** The results of RNA-seq, ATAC-seq and ChIP-seq of H3K27me3, H3K27ac and H3K4me4 around this region.

We next explored the 3D genome around the *FCGR3A* (Fc gamma receptor III A, also known as CD16), which is a dominant functional regulator in primary monocytes. The CD16 positive monocytes played pivotal immune surveillance functions in central nervous system and the upregulated FCGR3A is also related to the activation of microglial phagocytic capacity in neuroinflammation. A summary of the characteristics of this region is provided in **Figure 6**, including the insulation score values (**Figure 6A**) and the presence of loops (**Figure 6B**) and chromatin interactions (**Figure 6C**) in the six samples around the CD16-encoding gene as well as the results of RNA-seq, ATAC-seq and ChIP-seq analyses of H3K27me3, H3K27ac and H3K4me4 around this region (**Figure 6D**).

There were many SLE susceptibility-related SNPs in the region, among which we used rs403016 as a landmark, which is located in the coding region of the *FCGR3A* gene and has been reported to be associated with an increased risk of developing SLE. In our primary monocyte 3D structure maps, this SNP was shown to be located near a TAD boundary on chromosome 1q23 (**Figure 6A**). The low insulation score of the rs403016 region indicated that this region contained a TAD boundary. Considering that this value varied among the primary samples, we inferred that TAD boundary sliding may have occurred. Despite the conservation of the separation of chromatin interactions in this region on a large scale in both the cell lines and primary samples (**Figure 6A**), the contact maps varied near this region (**Figure 6C**), which was more meaningful.

In the *FCGR3A* promoter region (around rs403016), the loop distribution in the SLE-2 sample was similar to that in the THP1 cell line, while it differed considerably from those in the other three primary samples. In particular, the SLE-2 sample presented an increased interaction loop around the *FCGR3A* promoter, while the SLE-1 sample did not show this loop (**Figures 6B, C**). According to GeneHancer database, an enhancers (chr1:161,590,591-161,600,917) of *FCGR3A* had been identified by eQTLs, eRNA_co-expression assay (**Figure S12**). This enhancer overlapped the right side of varied loop (left side: 161.52M-161.53M, right side: 161.60M-161.61M), which bridged the enhancer to the promoter region of *FCGR3A*. Accordingly, the expression of *FCGR3A* was highest in the SLE-2 sample and lowest in SLE-1 (RNA-seq results in **Figure 6D**, and validated in qRT-PCR in **Figure S1E**) without significant differences according to ATAC-seq and ChIP-seq (**Figure 6D**), which could be attributed to the diverse loop to the upstream enhancer.

## Discussion

As yet the whole-genome 3D organization of primary human monocytes has not been thoroughly revealed. To fill this gap, herein we present 3D genomic maps of monocytes from peripheral blood samples collected from four individuals. Our Hi-C maps reach 5 kb resolution with high quality, which ensures the subsequent analysis is credible.

Through careful comparative analysis, we found that the primary monocyte samples showed roughly similar chromatin landscapes in terms of chromatin interaction frequencies, interaction matrices, A/B compartments and TAD boundaries, and few consistent patterns were found between the SLE patients and healthy controls. The primary samples displayed a highly conserved structural pattern of genome organization despite the different autoimmune statuses of their donors; even though two of the samples came from SLE patients, they all consisted of normal somatic cells with no deficiency. These negative results further confirmed that chromatin undergoes significant changes in high-level structures only when major structural changes, such as balanced translocations or deletions of large regions, occur with chromosomes. The observed conservation of advanced structures was consistent with biological rules and reflects a stable structural pattern of immune cells at different stages of autoimmunity.

In the present study, Hi-C data from the human monocyte cell line THP1 were also reanalyzed as a reference. The results showed that the 3D high-resolution maps of THP1 cells significantly differed from those of our primary monocyte samples according to unbiased assessment based on APA and comparative and correlation analyses of multiple Hi-C datasets. A previous investigation involving capture Hi-C was performed to compare the commonalities and differences in promoter interactions between CD34^+^ hematopoietic progenitor cells and the human B cell line GM12878, and the results indicated that alternate long-range interactions determined differential transcription programs in different cell types (31). The engineered THP-1 cell line, derived from the peripheral blood of an individual with acute monocytic leukemia, is usually used as a model to study the modulation of monocytes and macrophage functions (32). The divergence of the 3D chromatin structure of this cell line from that of primary monocytes is not surprising because THP1 cells are aneuploid cells; however, these differences could reduce the validity of THP1-based analysis. As shown in the correlation analysis of **Figure 3**, we found that in most of the genome region, the degree of variation between the 4 different individuals is similar to that of the two biological repeats of THP1 cells. This result also indicated that the primary monocytes are highly conserved in 3D genome.

Our 3D chromatin maps also showed significant diversity in the HLA region among individuals. The chromatin interactions around HLA-DQ and HLA-DR differed mostly among different primary monocyte samples and were associated with nearby gene expression. Genetic variation in intergenic regions of the HLA MHC-II locus is associated with multiple autoimmune diseases (33). Recent evidence has shown that significant diversity in histone modifications and super enhancer (SE) interactions within HLA-DR/DQ promoters or intergenic regions might dynamically contribute to SLE morbidity (34) or regulate the complexity of immune responses between individuals (35). Our findings indicate the underlying regulation of chromatin interactions around the HLA region, and the results were consistent with previous reports in B or T cells, while considering cell-type specificity, we firstly provide evidence of potential 3D regulation pattern in primary monocytes.

In this work, additional genotyping data showed that SLE-1 presented homozygosity of HLA-DRB1 (*15:01:01) and HLA-DQB1 (*06:02:01). Considering that SLE-1 presented the strongest chromatin interactions among the four studied individuals, we speculated that a relationship exists between the genotype and the strong chromatin interactions of these HLA regions. HLA-DRB1*15:01/DQB1*06:02 has been identified as the strongest classical SLE susceptibility-related allele in individuals of European, African, and Hispanic ancestries (36–38). Haplotypes bearing DRB1*1501/DQB1*0602 are key determinants of autoantibody production and disease susceptibility in human SLE (39). Great interest has been focused on the DRB1*1501/DQB1*0602 haplotype, which confers risk of autoimmunity resulting from changes in the epigenome (40). Therefore, it is reasonable to consider that the DRB1*1501/DQB1*0602 haplotype can potentially be therapeutically targeted by altering the 3D architecture to regulate the immune response in SLE pathogenesis.

We found that the *FCGR3A* was located at a variable TAD boundary and that the dynamics of chromatin loops might modulate CD16 expression. According to the surface expression of CD16 (*FCGR3A*), monocytes can be grouped into three subpopulations (41). The proportion of CD16-positive monocytes is tightly linked with certain autoimmune diseases (42–44), and many susceptibility-related SNPs around these regions have been validated in population studies (45, 46). Previous studies have explored the epigenetic regulatory mechanisms of CD16 in monocytes or other immune cells (47, 48). Here, we present the first evidence of chromatin loop-mediated regulation in this region based on the observed variation in the TAD boundary in the *FCGR3A* gene region, which indicated that the expression of CD16 was dominated by chromatin interactions. The identified enhancer loop regulations in *FCGR3A* suggested potential therapeutic target in autoimmune neurological diseases.

Nevertheless, taken together, our results indicate both the stability and variability of high-resolution chromatin interaction maps among human primary monocytes. The detailed 3D genomic landscape obtained in this work reveals potential regulatory functions related to monocytes. Our work highlights the complex interplay of the epigenetic and spatial 3D chromatin changes that are necessary to regulate gene expression and potentially mount an effective immune response. However, further functional experiments will be required for the validation of this work. The role of the 3D architecture in innate immunity is a topic of ongoing investigation.

## Data Availability Statement

The datasets presented in this study can be found in online repositories. The names of the repository/repositories and accession number(s) can be found in the article/**Supplementary Material**.

## Ethics Statement

The studies involving human participants were reviewed and approved by the Ethics Committee of Shandong University in China. The patients/participants provided their written informed consent to participate in this study.

## Author Contributions

YZ designed the study and revised the manuscript. YX and XWL performed the research and wrote the manuscript. WM and CM analyzed the data. YJ and BC collected clinical samples. SH and YG sorted CD14^+^ primary monocytes. HS and XL performed genotyping of HLA region. LW assisted in data transmission and analysis. All authors have reviewed and approved the manuscript.

## Funding

This work was supported by grants from the National Natural Science Foundation of China (82171665, 31370897), the Natural Science Foundation of Shandong Province (ZR2020MH168) and the Key Technology Research and Development Program of Shandong Province (2018GSF118113).

## Conflict of Interest

TL was employed by the company Annoroad Gene Technology.

The remaining authors declare that the research was conducted in the absence of any commercial or financial relationships that could be construed as a potential conflict of interest.

## Publisher’s Note

All claims expressed in this article are solely those of the authors and do not necessarily represent those of their affiliated organizations, or those of the publisher, the editors and the reviewers. Any product that may be evaluated in this article, or claim that may be made by its manufacturer, is not guaranteed or endorsed by the publisher.

## Supplementary Material

Supplementary Figure 1 Quality control of RNA-seq. **(A)** The heatmap of Pearson correlation matrix of genome-wide FPKMs values within samples. **(B)** The distance and hierarchical clustering of the four samples. **(C)** The volcanos plot of difference analysis between SLE group and CTR group, under the cut off value of |log2FC|>1.5 and padj < 0.05, there were 74 genes significant upregulated and 124 genes downregulated in SLE group. **(D)** The heatmap of expression (performed z-scale by each gene) of significant genes of C (74 up and 124 down). **(E)** The validation RNA-seq by qRT-PCR in the identical samples. We selected six genes (ACTB, GAPDH, FCGR3A, STAT3, FLT3 and CXCL8) and the left heatmap indicated their expression value (FPKM) in RNA-seq. The right heatmap shown their quantitative results(2^-deltaCt^) by qRT-PCR compared to the inner reference of GAPDH.

Supplementary Figure 2 Quality control of ATAC-seq. **(A)** The genome wide normalized peaks distribution of the four samples. **(B)** The heatmap of Pearson correlation matrix (upper) or Spearman correlation matrix (lower) of the four samples. **(C)** The normalized reads density distribution in gene profile of the four samples.

Supplementary Figure 3 Quality control of ChIP-seq. **(A)** The gene body normalized reads (RPKM) density plot of the nine ChIP-seq sample. **(B)** The heatmap of correlation matrix of normalized reads within the nine samples. **(C)** The gene body normalized reads (RPKM) heatmap of the nine ChIP-seq samples.

Supplementary Figure 4 The trans-interactions map of THP1 cell and primary monocyte. **(A)** Heatmap of all cis and trans interactions in genome wide. **(B)** The average intensity of interactions within or inter chromosomes. **(C)** The detailed interactions in chr9 to chr11 indicated rearrangement (blue box) between 9p and 11q in THP1-1 and THP1-2. **(D)** The circos plot of all significant trans interactions in genome wide.

Supplementary Figure 5 The relative contact probability (RCP) in all chromosomes. The full landscape of.

Supplementary Figure 6 Genome-wide all by all Hi-C interaction minus. From Delta Hi-C, it was nearly no difference in SLE group and CTR group in a comparative large scale (1M resolution).

Supplementary Figure 7 A/B compartment correlation and switch rate stat. A/B compartment between SLE group and CTR group were similar and had low switch Rate.

Supplementary Figure 8 The MD plot of comparison in all chromosomes. The full landscape of (upper panel).

Supplementary Figure 9 The difference matrix between primary monocytes and THP1 in all chromosomes. The full landscape of (lower panel).

Supplementary Figure 10 The correlation matrix between the six samples in all chromosomes. The full landscape of.

Supplementary Figure 11 The genotyping in HLA region.

Supplementary Figure 12 The evidence of enhancer regulation of FCGR3A. The loops of the six samples were the same as and the lower panel indicated the annotation of enhancer from GeneHancer database.

Supplementary Data Sheet 1 Full results of RNA-seq.

Supplementary Data Sheet 2 Full results of ATAC-seq.

Supplementary Data Sheet 3 Full results of ChIP-seq.
