# Supplementary figures and images for: Capturing 3D Chromatin Maps of Human Primary Monocytes: Insights From High-Resolution Hi-C
Source: Front Immunol. 2022 Mar 3;13:837336. doi: 10.3389/fimmu.2022.837336 (PMC8927851; doi:10.3389/fimmu.2022.837336)

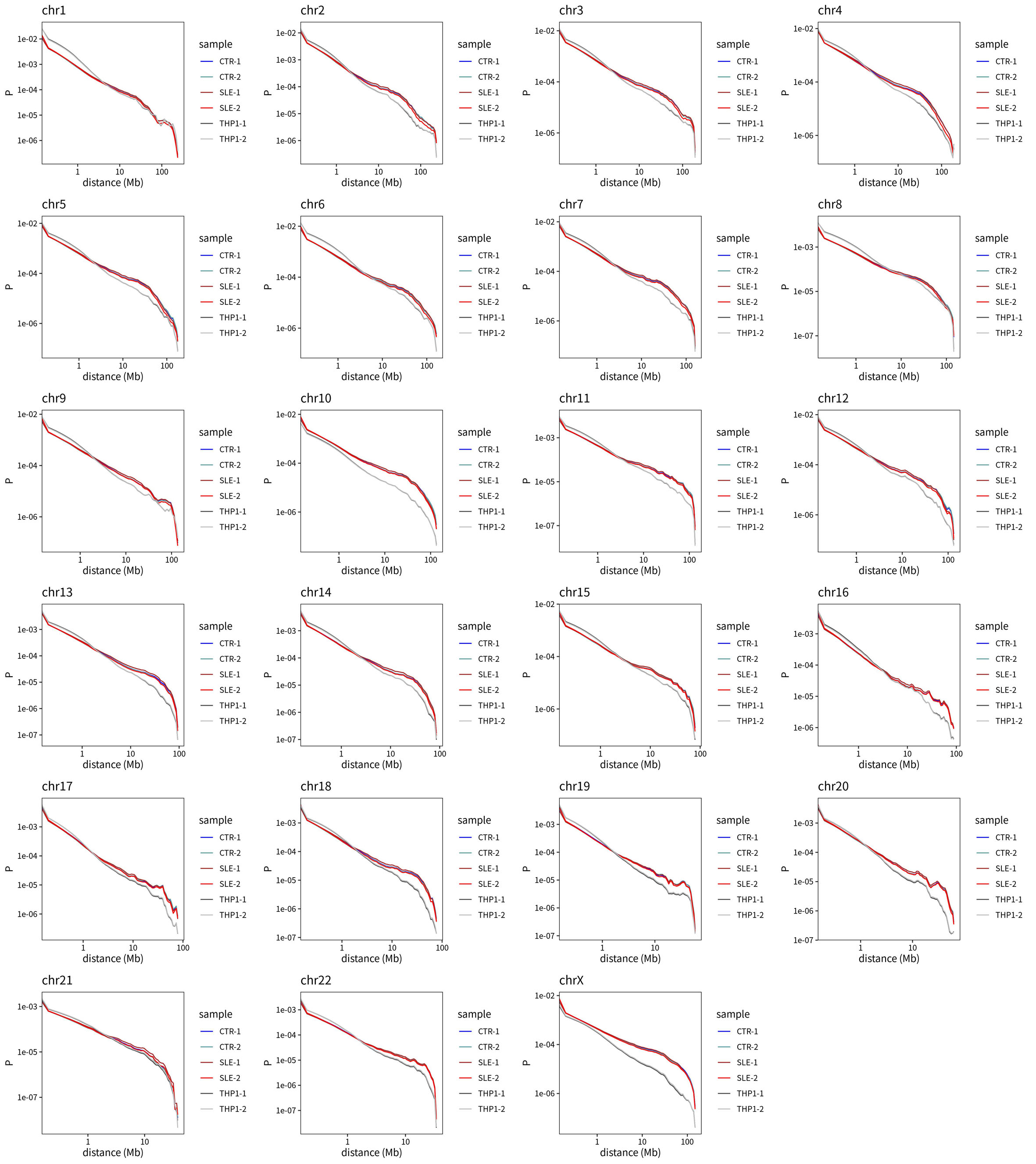

Supplement: Supplementary Figure 5 — The relative contact probability (RCP) in all chromosomes. The full landscape of. [file Image_5.jpeg]

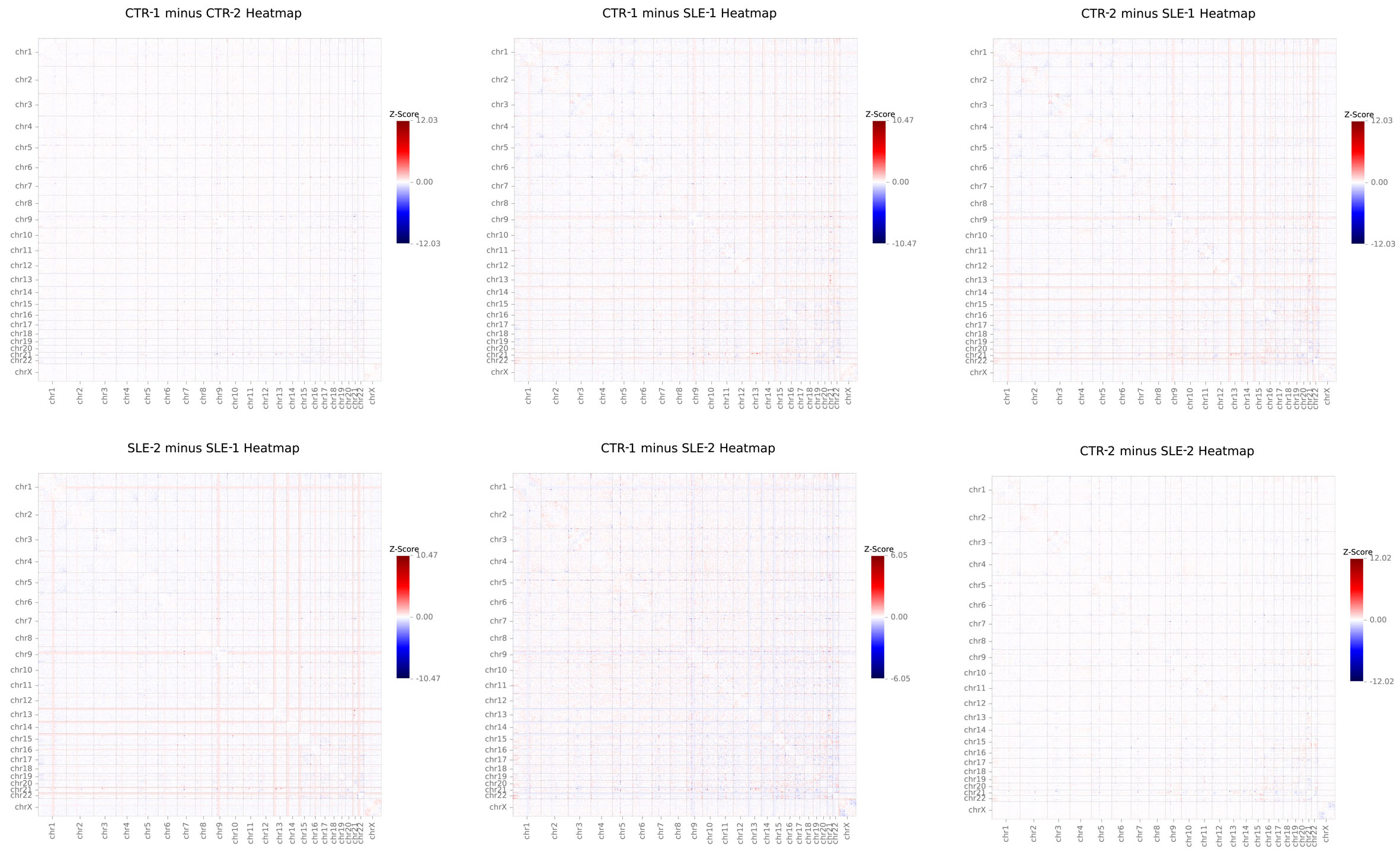

Supplement: Supplementary Figure 6 — Genome-wide all by all Hi-C interaction minus. From Delta Hi-C, it was nearly no difference in SLE group and CTR group in a comparative large scale (1M resolution). [file Image_6.jpeg]

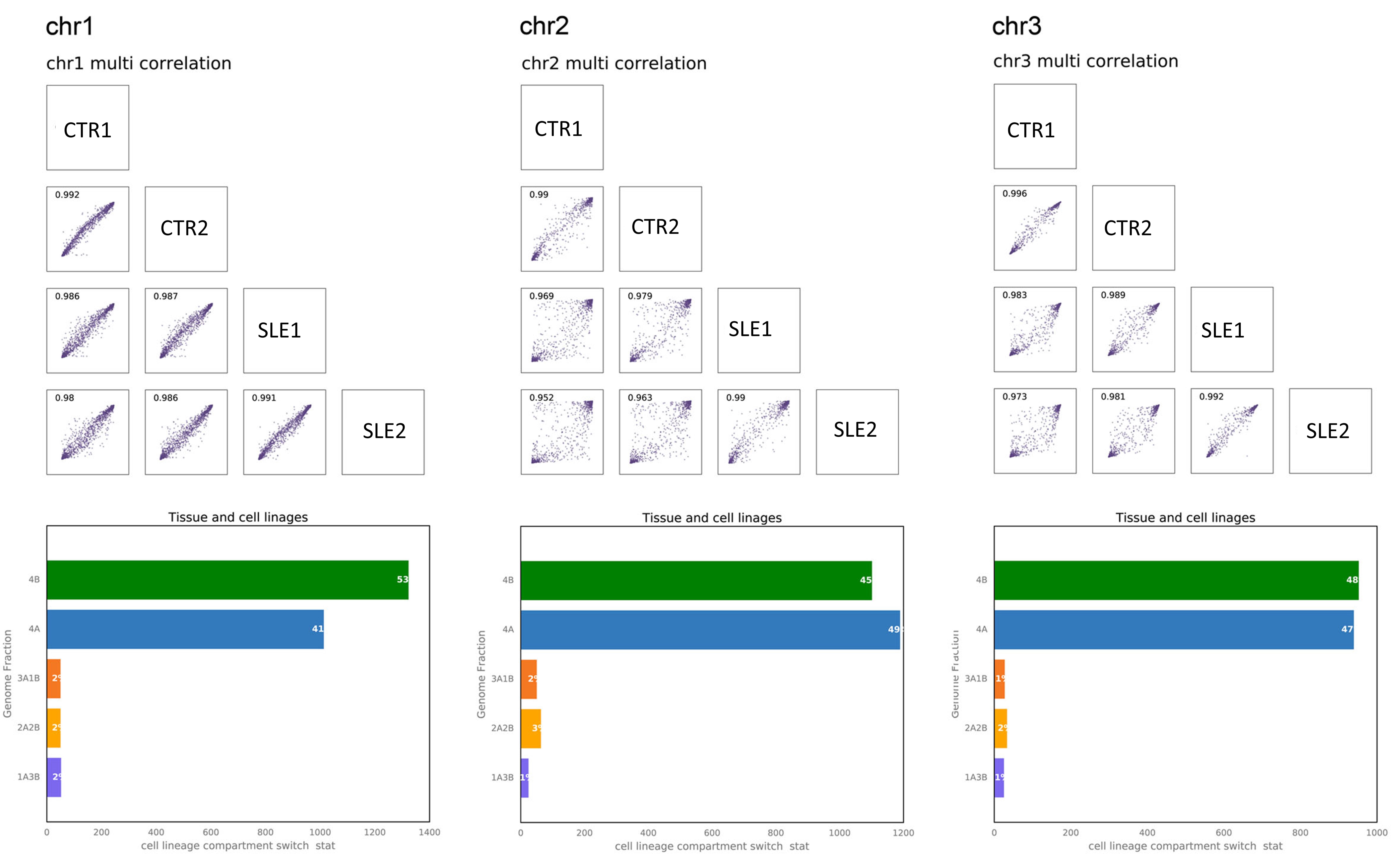

Supplement: Supplementary Figure 7 — A/B compartment correlation and switch rate stat. A/B compartment between SLE group and CTR group were similar and had low switch Rate. [file Image_7.jpeg]

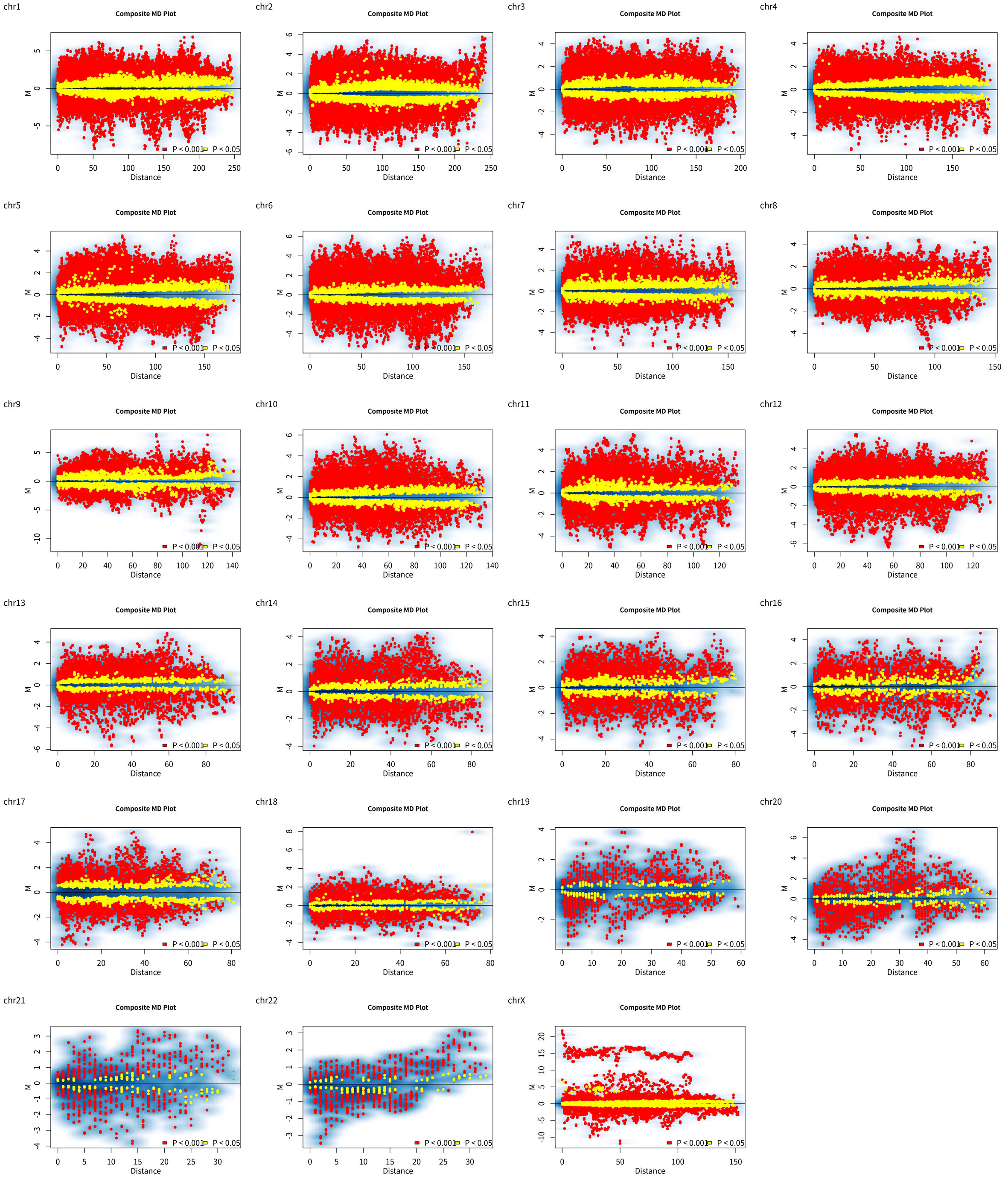

Supplement: Supplementary Figure 8 — The MD plot of comparison in all chromosomes. The full landscape of (upper panel). [file Image_8.jpeg]

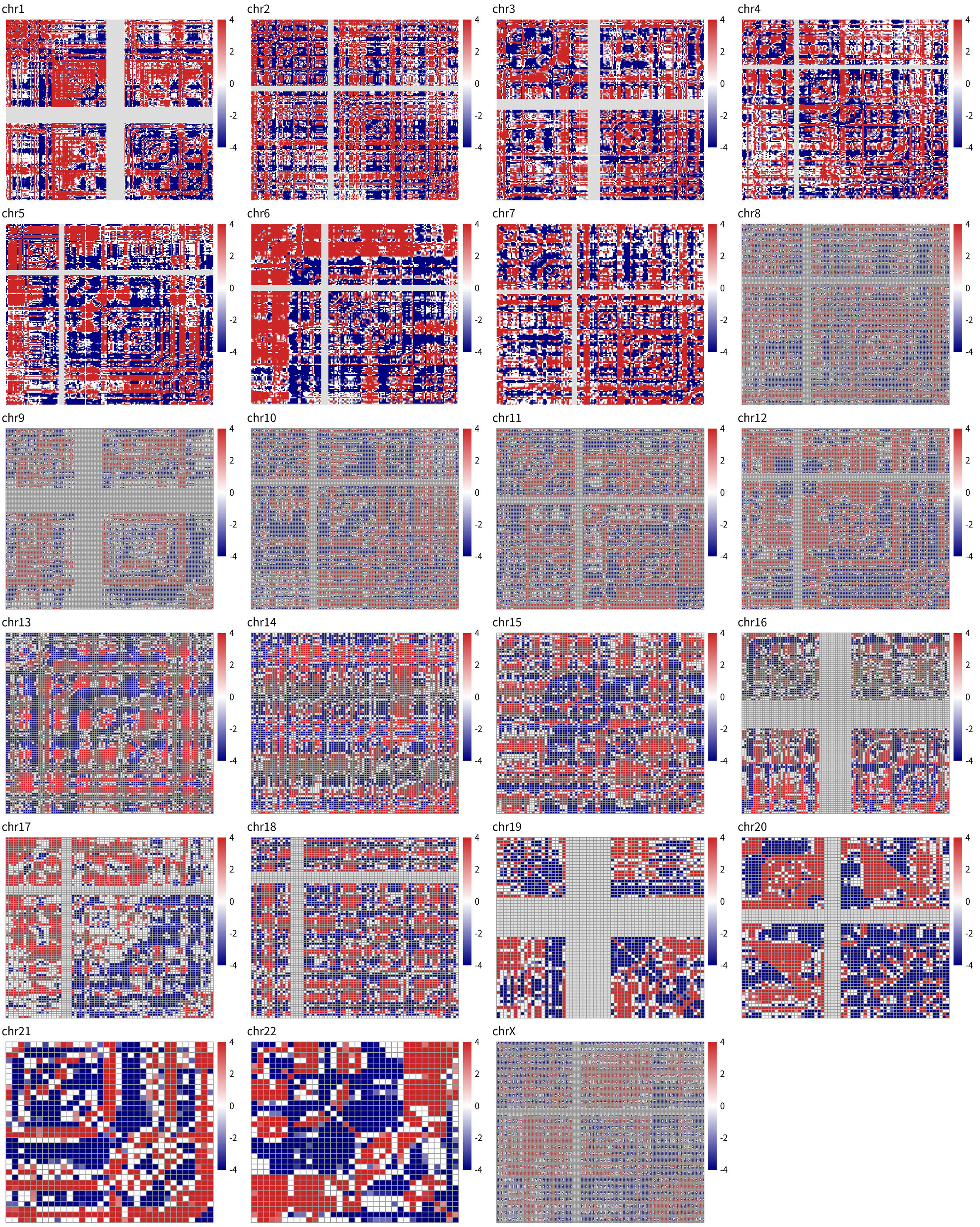

Supplement: Supplementary Figure 9 — The difference matrix between primary monocytes and THP1 in all chromosomes. The full landscape of (lower panel). [file Image_9.jpeg]

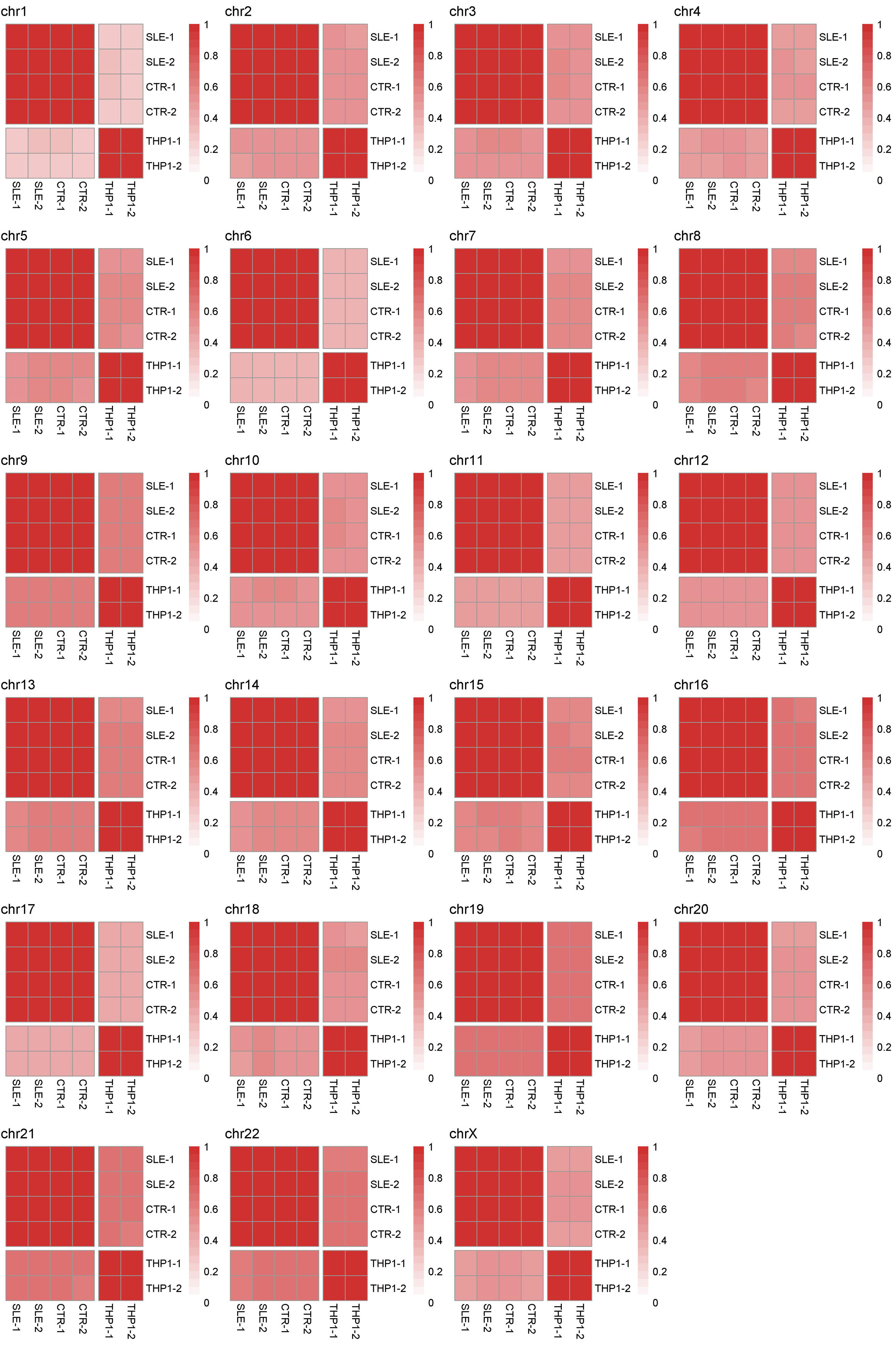

Supplement: Supplementary Figure 10 — The correlation matrix between the six samples in all chromosomes. The full landscape of. [file Image_10.jpeg]

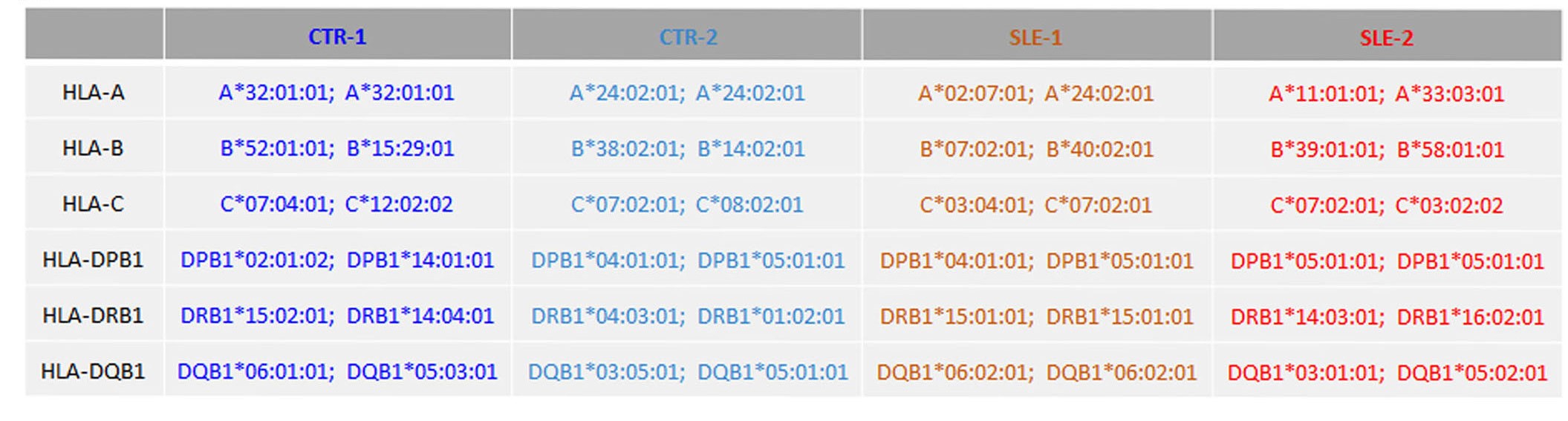

Supplement: Supplementary Figure 11 — The genotyping in HLA region. [file Image_11.jpeg]

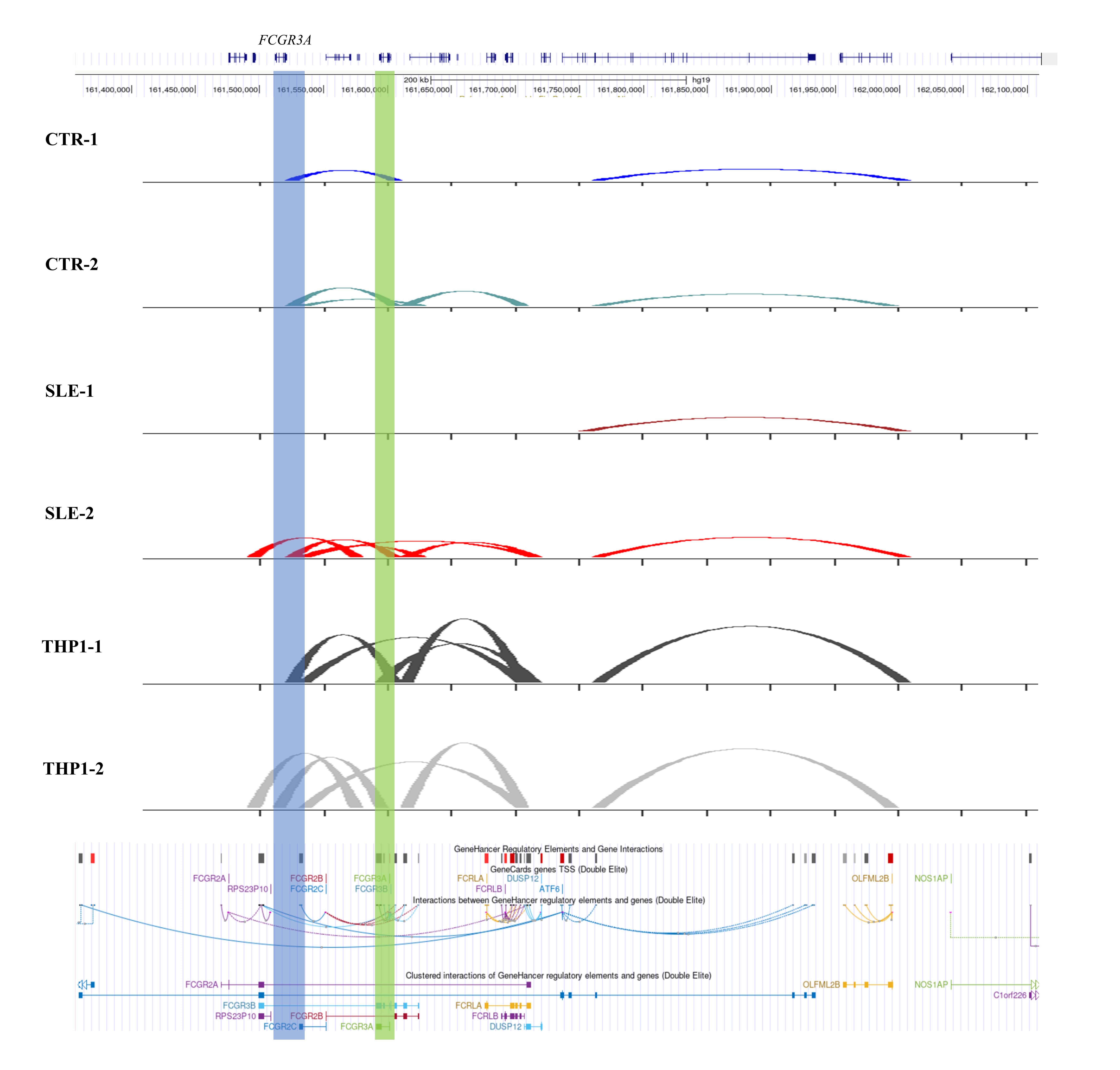

Supplement: Supplementary Figure 12 — The evidence of enhancer regulation of FCGR3A. The loops of the six samples were the same as and the lower panel indicated the annotation of enhancer from GeneHancer database. [file Image_12.jpeg]
