# Supplementary material for: Capturing 3D Chromatin Maps of Human Primary Monocytes: Insights From High-Resolution Hi-C
Source: Front Immunol. 2022 Mar 3;13:837336. doi: 10.3389/fimmu.2022.837336 (PMC8927851; doi:10.3389/fimmu.2022.837336)
Supplement: Supplementary file 13 [file Table_1.docx]

sTable1, The statistics of Hi-C results of the four primary monocytes

| Sample | CTR-1 | CTR-2 | SLE-1 | SLE-2 |
| --- | --- | --- | --- | --- |
| Clean Paired-end Reads | 3,008,455,392 | 3,027,459,893 | 3,402,672,695 | 2,943,082,507 |
| Unique Mapped Ratio (%) | 65.78 | 65.11 | 67.12 | 65.68 |
| Unique Mapped Paired-end Reads | 1,978,928,164 | 1,971,134,862 | 2,284,021,273 | 1,932,904,515 |
| Valid Paired-end Reads | 1,326,350,377 | 1,248,336,917 | 1,607,666,898 | 1,321,125,782 |
| Valid Rate (%) | 67.02 | 63.33 | 70.39 | 68.35 |
| Total Number of called loops | 5345 | 7315 | 5178 | 11859 |
