# Supplementary material for: Capturing 3D Chromatin Maps of Human Primary Monocytes: Insights From High-Resolution Hi-C
Source: Front Immunol. 2022 Mar 3;13:837336. doi: 10.3389/fimmu.2022.837336 (PMC8927851; doi:10.3389/fimmu.2022.837336)
Supplement: Supplementary file 14 [file Table_2.docx]

sTable2, Switch Genes KEGG Enrichment

| Name | p |
| --- | --- |
| Neuroactive ligand-receptor interaction | 7.93E-06 |
| Drug metabolism - cytochrome P450 | 0.00101549 |
| Drug metabolism - other enzymes | 0.001181196 |
| Hepatocellular carcinoma | 0.001378447 |
| Metabolism of xenobiotics by cytochrome P450 | 0.001247673 |
| Chemical carcinogenesis | 0.002066732 |
| Fluid shear stress and atherosclerosis | 0.002271892 |
| Phenylalanine, tyrosine and tryptophan biosynthesis | 0.003416063 |
